# Autoerythrocyte Sensitization Syndrome Treated with Kallikrein Inhibitor

**DOI:** 10.4274/tjh.48265

**Published:** 2013-03-05

**Authors:** Sevgi Gözdaşoğlu

**Affiliations:** 1 Retired Professor of Pediatrics, Hematology

**To the Editor,**

I read the letter related to autoerythrocyte sensitization syndrome in a 7-year-old boy published in the Turkish Journal of Hematology [1]. I would like to remark on a few points that were not mentioned in that study. Autoerythrocyte sensitization syndrome was first described in 1972 by Karaca et al. from Turkey. They reported in detail on all characteristics in 2 patients, 19- and 23-year-old women [2,3], and a girl having severe emotional problems along with Gardner-Diamond syndrome was reported as the first childhood case in 1974 in our department [4].

The noteworthy characteristic of these ecchymoses is that they begin with local itching, burning, or pain before the lesions appear. Skin lesions are found to be correlated with periods of increased psychic stress and these patients are prone to hysteria, masochism, depression, and anxiety. The relationship between the syndrome and an underlying psychiatric disorder has been clearly emphasized. Although the etiological component of emotional stress in the formation of the lesions is not clear, the neuroimmune system is postulated as a mediator in the appearance of lesions. Gastrointestinal bleeding, epistaxis, hematuria, abdominal pain, diarrhea, nausea and vomiting, syncopal attacks, chest pain, headache, and menometrorrhagia are other findings or complaints in these patients [5,6].

The pathogenesis of autoerythrocyte sensitization syndrome remains unclear.

Agle and Ratnoff identified polypeptides similar to bradykinin occurring in subcutaneous tissues in these patients [7,8]. Leiba et al. reported the possible role of bradykinin in the pathogenesis of the syndrome (DNA sensitization) [9].The kinin-kallikrein system is complex, with several bioactive peptides that are formed in many different compartments. This system plays a role in inflammation, coagulation, sodium homeostasis, sensations of pain, cardioprotective effects of proconditioning, and control of blood pressure [10]. Regulation in venous capillary tonus by fluctuations in the kinin-kallikrein system is suggested [6].

All of these studies suggest to us the treatment of the syndrome with bradykinin inhibitor. In our case, a 20-year-old married woman suffering from nausea, vomiting, abdominal pain, fatigue, and ecchymoses around the lower part of her extremities was admitted to the Department of Gastroenterology of the Ankara University School of Medicine. Characteristic skin lesions were present on the ventral surfaces of her legs. These lesions were painful, edematous, and of 2 to 6 cm in diameter. Physical examination was otherwise within normal limits. Routine laboratory tests and coagulation studies were normal. The skin lesions started spontaneously or following minimal trauma. They were characterized by itching and pain, followed by edematous ecchymoses. Intradermal injection of 0.1 mL of 60% suspension of washed red blood cells from the patient reproduced these characteristic skin lesions while the serum and plasma of the patient and saline were ineffective. The tests were repeated several times and found to be positive. She was diagnosed with autoerythrocyte sensitization syndrome and treated with kallikrein inhibitor aprotinin at 2000 IU/kg IV in 8 h [11]. After the treatment, all skin lesions disappeared and the patient received psychological support. The beneficial results obtained with kallikrein inhibitor in this case is suggestive of an important role of the kinin–kallikrein system on the pathogenesis of autoerythrocyte sensitization syndrome.

**Conflict of Interest Statement**

The authors of this paper have no conflicts of interest, including specific financial interests, relationships, and/ or affiliations relevant to the subject matter or materials included.

## REPLY

**Dear Editor,**

I thank the author for her polite contribution. Notwithstanding, in the first paragraph, according to her declaration sentence of “Autoerythrocyte sensitization syndrome was first described in 1972 by Karaca et al. from Turkey”, it is understood as is the syndrome had been first defined in Turkey. However, it was first defined in 1955 by Gardner and Diamond, not in Turkey. Thus, the sentence seems to require correction in order not to be misinterpreted, say “Autoerythrocyte sensitization was first defined in 1955 by Gardner and Diamond, and the first case from Turkey was defined in 1972 by Karaca et al. from Turkey”.

I also thank you for your polite message and admirable concern. 

**Best wishes**

**Mesut Okur, MD**
